# Impact of DnaK on protein expression and deamidation modification in *Cronobacter sakazakii*: insights from proteomic analysis

**DOI:** 10.1128/MRA.00516-23

**Published:** 2023-09-25

**Authors:** Ping Lu, Juan Xue, Xi Chen, Xuemeng Ji

**Affiliations:** 1 Tianjin Key Laboratory of Ophthalmology and Visual Science, Tianjin Eye Institute, Tianjin Eye Hospital, Tianjin, China; 2 Nankai University Affiliated Eye Hospital, Nankai University, Tianjin, China; 3 Clinical College of Ophthalmology, Tianjin Medical University, Tianjin, China; 4 Institute of Infection and Immunity, Taihe Hospital, Hubei University of Medicine, Shiyan, China; 5 School of Medicine, Nankai University, Tianjin, China; University of Guelph, Guelph, Guelph, Canada

**Keywords:** *Cronobacter sakazakii*, proteomics, DnaK, chaperone, deamidation

## Abstract

This study explores the influence of DnaK on intracellular proteins in *Cronobacter sakazakii*, a Gram-negative bacterium known for causing infections, particularly in neonates. Proteomic and post-translational modification analyses were performed, revealing a potential link between DnaK and protein deamidation.

## ANNOUNCEMENT

*Cronobacter sakazakii* is a Gram-negative bacterium known for its ability to cause meningitis and sepsis in individuals of various age groups, with neonates being particularly susceptible (1–3). DnaK, a ubiquitous molecular chaperone conserved in bacteria, has been linked to bacterial acid tolerance, desiccation tolerance, and pathogenicity (4). This study is aimed to examine the influence of *dnaK* on intracellular proteins by conducting proteomic and post-translational modification analyses.

The *C. sakazakii* ATCC BAA-894 strain, recognized internationally as a reference strain (5), had been preserved in glycerol tubes at −80℃. Before conducting the experiments, the strain was revived by culturing it overnight in Luria-Bertani (LB) broth at 37℃ with shaking. To generate an in-frame *dnaK* deletion mutant, the pCVD442 suicide vector was utilized, which was amplified using *Escherichia coli* S17 **λ** pir. The previously established pCVD442 suicide vector method was employed to create the in-frame deletion mutants (6). The successful deletion was confirmed by PCR using outside primers and sequencing. Both the wild-type strain and its *dnaK* deletion mutant were employed for extracting proteins and subsequent proteomic analysis.

Following an overnight culture in LB medium at 37℃ with shaking, the wild-type and knockout strains were harvested via centrifugation. The collected cells were washed with PBS buffer and then combined with a protease inhibitor cocktail (Beyond, China) and PBS. Subsequently, the cells were lysed using ultrasonic waves in an ice bath for 30 min, and the resulting lysate was clarified utilizing a microporous filter with a pore size of 0.22 µm. To determine the protein concentration, a BCA assay kit (Thermo, USA) was employed. A mixture comprising 50 µg of protein, 1 µL of 200 mM tris(2-carboxyethyl)phosphine (Aladdin, China), 1 µL of 500 mM iodoacetamide (Aladdin, China), and a total volume of 100 µL with 6M guanidine hydrochloride (Aladdin, China) was prepared. The mixture was incubated in darkness at room temperature for 40 min, and the protein was purified using a 10 kD ultrafiltration tube (Millipore, USA). Subsequently, the protein was digested overnight at 37°C using 2 µg of recombinant trypsin (from Promega, USA) in the ultrafiltration tube (7). The resulting filtrate was obtained through centrifugation, followed by desalting using a desalting column (Millipore, USA). Finally, mass spectrometry analysis was performed after adding 0.1% formic acid (Aladdin, China) to the desalted sample.

The generated peptides were analyzed using a Q Exactive Plus mass spectrometer (Thermo, USA), an instrument with an atmospheric pressure ionization (API) source with S-lens ion optics technology. The gradient length was 60 min. The mass spectra were searched in the UniProt database (Taxon ID 290339, downloaded on 10 December 2021) using Maxquant software (Version 2.1.0.0). The analysis parameters for Maxquant included carbamidomethylation of cysteine as a fixed modification and oxidation of methionine and deamidation as variable modifications. Protein and peptide identifications were determined with a maximum false discovery rate (FDR) of 1.0%, and label-free quantification in MaxQuant was normalized by the median. The proteomics data underwent bioinformatics analysis, including Gene Ontology (GO) and Kyoto Encyclopedia of Genes and Genomes (KEGG) pathway analysis, utilizing the DAVID online database. During the statistical analyses, a correction factor of 5% false discovery rate was applied for multiple hypothesis testing. The reported proteins exhibited significant differences in abundance after the FDR correction. For the significance assessment, a *P*-value (Student’s *t*-test) less than 0.05 was used, and a fold change exceeding 2 was considered statistically significant. The statistical analyses were conducted using GraphPad Prism software (Version 8.0).

A total of 1,448 proteins, equivalent to 32.7% of the total proteome, were successfully identified. Notably, the absence of DnaK led to a significant upregulation of 147 proteins, while eight proteins were found to be downregulated. Interestingly, the downregulated proteins did not display any notable enrichment in the GO and KEGG pathways (8). In contrast, the upregulated proteins exhibited significant enrichment in various biological processes, including cell division, protein folding, and peptidoglycan biosynthesis. Moreover, enrichment was observed in the cytoplasmic cellular component, indicating the involvement of these proteins in essential intracellular processes. Functionally, these upregulated proteins were found to be associated with ATPase activity, manganese ion binding, and ATP binding. Further KEGG analysis revealed significant upregulation in crucial metabolic pathways, including amino acid biosynthesis, secondary metabolite biosynthesis, antibiotic biosynthesis, lysine biosynthesis, and arginine biosynthesis. Collectively, these findings suggest that *C. sakazakii* employs a compensatory mechanism by upregulating protein expression in response to protein stress caused by the knockout of *dnaK*.

A previous study revealed that *p*-coumaric acid treatment in *C. sakazakii* led to deamidation and a growth disadvantage (8), indicating a potential impact of protein deamidation on bacterial phenotypes. To investigate the potential protein stress caused by *dnaK* knockout, we performed protein deamidation quantification using MaxQuant. Specifically, we utilized set deamidation (NQ) as a variable modification during the search process. Seventy-seven exhibited deamidation modifications. Interestingly, 34 proteins (44%) showed a significant increase in abundance following *dnaK* knockout, indicating their potential involvement in the cellular response to *dnaK* depletion. The subsequent GO and KEGG analyses of these proteins demonstrated significant enrichment in molecular functions associated with rRNA binding as well as cellular components related to the cytoplasm. These findings suggest that the absence of *dnaK* may exert an influence on ribosome function, highlighting the potential role of *dnaK* knockout-induced protein stress.

## Data Availability

Proteomics datasets have been deposited to iProX (http://www.iprox.org/index) and can be accessed with the accession IPX0005932000 (URL: https://www.iprox.cn/page/project.html?id=IPX0005932000) (9, 10).
